# Array CGH Analysis of Paired Blood and Tumor Samples from Patients with Sporadic Wilms Tumor

**DOI:** 10.1371/journal.pone.0136812

**Published:** 2015-08-28

**Authors:** Leila Cabral de Almeida Cardoso, Lara Rodriguez-Laguna, María del Carmen Crespo, Elena Vallespín, María Palomares-Bralo, Rubén Martin-Arenas, Inmaculada Rueda-Arenas, Paulo Antonio Silvestre de Faria, GT-CSGP Working Group, Purificación García-Miguel, Pablo Lapunzina, Fernando Regla Vargas, Hector N. Seuanez, Víctor Martínez-Glez

**Affiliations:** 1 Genetics Division, Instituto Nacional de Câncer, Rio de Janeiro, Brazil; 2 Section of Functional and Structural Genomics, Institute of Medical and Molecular Genetics (INGEMM)-IdiPAZ, Hospital Universitario La Paz, Madrid, Spain; 3 CIBERER, Centro de Investigación Biomédica en Red de Enfermedades Raras, Madrid, Spain; 4 Pathology Division, Instituto Nacional de Câncer, Rio de Janeiro, Brazil; 5 Service of Pediatric Oncohematology-IdiPAZ, Hospital Universitario La Paz, Madrid, Spain; 6 Section of Clinical Genetics, Institute of Medical and Molecular Genetics (INGEMM)-IdiPAZ, Hospital Universitario La Paz, Madrid, Spain; 7 Genetics Department, Universidade Federal do Rio de Janeiro, Rio de Janeiro, Brazil; 8 Birth Defects Epidemiology Laboratory, Fundação Oswaldo Cruz, Rio de Janeiro, Brazil; University of Bristol, UNITED KINGDOM

## Abstract

Wilms tumor (WT), the most common cancer of the kidney in infants and children, has a complex etiology that is still poorly understood. Identification of genomic copy number variants (CNV) in tumor genomes provides a better understanding of cancer development which may be useful for diagnosis and therapeutic targets. In paired blood and tumor DNA samples from 14 patients with sporadic WT, analyzed by aCGH, 22% of chromosome abnormalities were novel. All constitutional alterations identified in blood were segmental (in 28.6% of patients) and were also present in the paired tumor samples. Two segmental gains (2p21 and 20q13.3) and one loss (19q13.31) present in blood had not been previously described in WT. We also describe, for the first time, a small, constitutive partial gain of 3p22.1 comprising 2 exons of *CTNNB1*, a gene associated to WT. Among somatic alterations, novel structural chromosomal abnormalities were found, like gain of 19p13.3 and 20p12.3, and losses of 2p16.1-p15, 4q32.5-q35.1, 4q35.2-q28.1 and 19p13.3. Candidate genes included in these regions might be constitutively (*SIX3*, *SALL4*) or somatically (*NEK1*, *PIAS4*, *BMP2*) operational in the development and progression of WT. To our knowledge this is the first report of CNV in paired blood and tumor samples in sporadic WT.

## Introduction

Wilms tumor (WT) is the most common cancer of the kidney in infants and children, accounting for 6 to 7% of all children neoplasms, with a prevalence of 1:10.000 affected individuals [1, 2]. This tumor originates from nephrogenic rests of pluripotent, potentially oncogenic, kidney precursors comprising non-differentiated blastemal cells, primitive epithelium structures and stromal components which present classical triphasic histology [3]. The average age of presentation is 42–47 months for children with unilateral WT and 30–33 months for bilateral WT [2]. Treatment, though differing between countries, is determined by clinical-pathologic stage and histological classification [4]. WT is frequently sporadic, occurring in only one member of a family or rarely affecting more than one (in familial WT).

WT has a complex etiology with genetic, constitutional and somatic factors playing dissimilar roles in its genesis and progression. This results in extensive genomic alterations including copy number variants (CNV) that may include oncogenes or tumor suppressor genes leading to dose-dependent, functional disruptions of gene expression. Identification of CNV regions may thus provide a better understanding of cancer development as well as insights for clinical management, diagnosis and therapeutic targets [5]. Although several tumors apparently show normal karyotypes regardless of stage and histopathology [6], the frequent loss of 11p13 in WAGR syndrome (Wilms tumor, aniridia, genital anomalies, mental retardation) led to the discovery of *WT1*, the first gene found in association with WT, with a relevant involvement in the development of kidney progenitor cells [7]. Germline missense mutations in *WT1* exon 8 or 9 are associated to Denys-Drash syndrome (pseudo- or true hermaphroditism, nephropathy, and genital anomalies with an estimated risk for WT higher than 90%) [8] while germline, point mutations in the *WT1* intron 9 donor splice site account for several cases of WT associated to Frasier syndrome (pseudohermaphroditism and progressive glomerulopathy) [9, 10]. Moreover, WT has been found to be associated to 5% of cases of Beckwith-Wiedemann syndrome (macroglossia, overgrowth, omphalocele, neonatal hypoglycemia, ear creases/pits, adrenocortical cytomegaly and renal abnormalities) but this condition, however, is unrelated to germline *WT1* mutations, but to 11p15.5 epigenetic alterations [4, 11]. In contrast, in sporadic, non-syndromic WT, tumor predisposition is usually not the result of *WT1* constitutive mutations [12].

Microarray Comparative Genomic Hybridization (aCGH) allows the identification, with high resolution, of CNV in tumor genomes which may identify biomarkers and genes involved in tumor progression [13]. aCGH studies in WT have identified whole, arm- and segmental- chromosome gains or losses and carried out comparative analyses of primary and relapsed tumors throughout their molecular evolution. They have also contributed to the understanding of genomic alterations of different histological profiles, their influence on prognosis, and identification of potential therapeutic gene targets [6, 14–17].

To date, aCGH studies in WT have only been performed in tumor tissue samples. In this work, we report molecular studies in paired, blood and tumor samples from 14 patients with sporadic WT, with the aim of identifying germline and somatic anomalies associated with this tumor. We herein describe a novel set of constitutional abnormalities and point to candidate genes that might be associated with WT development.

## Individuals, Materials and Methods

### Patients

We analyzed paired DNA samples of peripheral blood/fresh tumor tissue from 14 patients (10 males, 4 females) with histologically confirmed WT. Four patients presented additional clinical features, Patient 6, showed 5th finger clinodactyly, 2–3 syndactyly and alopecia areata, while Patient 13 showed a single cafe au lait spot, a frequent clinical traits not indicative of a syndromic condition. Patients 4 and 11 showed clearly dysmorphic features unrelated to any specific syndromic condition (Table 1).

**Table 1:** Main characteristics of patients and aCGH results.

Patients	Gender	Dx age (months)	Clinical/dysmorphic Features*	Histopathology	Tumor stage (SIOP)	Mutational screening**	Blood samples***	Tumor samples***
1	M	136		U; Bl	III			• arr1q21.1-q41(144995225–218587437)x3
								• arr11p13(24657785–41118849)x1 [*WT1*]
								• arr19p13.3(2598941–4148338)x1 [*GNG7*, *SLC39A3*, *SGTA*, *TLE6*, *TLE2*, *CELF5*, *NFIC*, *FZR1*, *HMG20B*, *GIPC3*, *DAPK3*, *MATK*, *EEF2*, *PIAS4*, *ZBTB7A*, *MAP2K2*]
2	M	90		U; Bl	III			• del: Chr18
								• arr1q21.1-q31.3(145009521–194370783)x3
3	F	62		U; Tri	I			• del: Chr22
								• arr17p13.2-p11. 2(0–17259610)x1[*TP53*]
4	M	50	Birth weight 3950g, height 53cm. Triangular face, 2–3 syndactyly.	B; Tri; FA; PLNR	V			• dup: Chr2,6,8,10,12,13,15,17
							• arr3p22.1 [chr3:41274533–41275414]x3 [*CTNNB1*]	• arr3p22.1 (41274533–41275414)x3 [*CTNNB1*]
								• arr20p12.3(6750630–6751620) x3 [*BMP2*]
5	M	36		U; Tri; FA	I	WTX (Blood and Tumor) ENST00000330258: c.1678C>T, g.14136C>T, R560W (rs200798538)		• Dup: Chr1q,7q,16p
								• del: Chr7p,10p,16q
								• arr 2p16.1-p15 (58944114–63817028)x1 [*REL*, *COMMD1*, *EHBP1*, *PAPOLG*, *PUS10*]
								• arr20p12.3(6750630–6751620)x3 [*BMP2*]
6	F	48	5th finger clinodactyly, 2–3 syndactyly, alopecia areata.	U; Tri	II			• dup: Chr8q,20
								• del: Chr7p,16q
								• arr1q21.1-q44(143745280–249250621)x3
							• 19q13.31 [chr19:44709104–44824025]x1 [*ZNF227*, *ZNF233*, *ZNF235*]	• arr19q13.31 (44709104–44824025)x1 [*ZNF227*, *ZNF233*, *ZNF235*]
7	M	42		U; Tri	II			• dup: Chr1q,12
8	M	61		B; Ep; PLNR	V	CTNNB1 (Tumor) ENST00000349496: c.121A>G, g.29797A>G, T41A (rs121913412)		• dup: Chr1q,6,12,16p
								• del: Chr16q
								• arr4q35.2-q28.1(127553960–190800944) x1 [*NEK1*]
								• arr11p13(32410163–32410439)x3 [*WT1*]
								• arr17q21.31-q25.3(44787865–81029941)x3[*TP53*]
9	M	67		U; Tri	I			• dup: Chr1q,6,7,10,12,X
								• del: Chr16q
								• arr11q22.1–24.3(99570424–127020920) x1
10	F	7		B; Tri	V			• dup: Chr1q,20
								• del: Chr21
								• arr1p22.2-p36.3(0–86615744) x1
11	F	35	Prominent metopic suture, round face, obesity, high stature, macrocephaly, anteverted nostrils, hypoplastic and inverted nipples, deep philtrum, folded ear lobes, scoliosis, clinodactyly, nevus, multiple cafe au lait spots, genu varus, short hands and feet, short fingers.	U; Tri	II		• arr20q13.3(50417421–50418192)x3 [*SALL4*]	• arr20q13.3(50417421–50418192)x3 [*SALL4*]
12	M	28		U; Tri	III	WTX (Tumor) ENST00000330258: c.437_438insT, g.12895_12896insT, V147Cfs*11		• dup: Chr8,12
								• arr19p13.3(616080–773663)x3 [*PALM*, *FSTL3*, *RNF126*, *FGF22*, *POLRMT*, *HCN2*]
13	M	25	Periumbilical single cafe au lait spot (+/- 4cm)	U; Bl	III	WT1 (Blood and Tumor) ENST00000379079: c.736C>T, g.43599C>T, R246*(rs121907909)	• arr2p21(45171857–45172687)x3 [*SIX3*]	• arr2p21(45171857–45172687)x3 [*SIX3*]
							• arr4p16.3(1806741–1807381) x3 [*FGFR3*]	• arr4p16.3(1806741–1807381) x3 [*FGFR3*]
14	M	56		U; St	II			• dup: Chr3,6,7,8,9,10,12
								• arr4q32.5-q35.1(169600832–191154276) x1 [*NEK1*]

M, male; F, female; Dx age, age at diagnosis in months; U, unilateral; B, bilateral; Tri, triphasic; Bl, blastemal; Ep, epithelial; St, stromal; FA, focal anaplasia; PLNR, perilobar nefrogenic rests; del, deletion; dup, duplication.

*Only clinical features from patients with constitutional alterations are showed.

** Results reported by Cardoso et al (2013).

*** aCGH coordinates are according to the GRCh37/hg19 genome. WT candidate genes from not previously reported segmental cryptic alterations are shown in brackets.

All tumor samples were collected from primary tumors from patients treated with the same neoadjuvant chemotherapy following the International Society of Paediatric Oncology (SIOP) WT 2001 trial protocol [18]. Age at diagnosis ranged from 7 to 136 months, with an average age at diagnosis of 53 months. Bilateral tumors were present in 3/14 (21.4%) patients with an average age at diagnosis of 39.34 months. Focal anaplasia was observed in 2/14 (14.3%) patients as well as perilobar nephrogenic rests (PLNR) in 2/14 (14.3%). Histological components varied, comprising 9/14 (64.3%) triphasic, 3/14 (21.4%) with blastemal predominance, 1/14 (7.14%) with an epithelial tumor, and 1/14 (7.14%) with a stromal tumor. Tumors were classified according to stage: I (3/14; 21.4%), II (4/14; 28.6%), III (4/14; 28.6%), and V (3/14; 21.4%) according to the SIOP clinic-pathologic criteria. Stage IV tumors were not present in this series.

This study was approved by the *Instituto Nacional de Câncer* (Brazil) Ethics Committee and the Clinical Research Ethics Committee of *Hospital La Paz* (Madrid, Spain), and was performed in accordance to the ethical standards prescribed in the 1964 Helsinki Declaration. All parents or guardians on behalf of the children enrolled in this study were given the option to accept or reject participation. Following acceptance, a signed, informed consent was provided. Informed consents are kept in patients' files with copies provided to parents/guardians. Details that might disclose the identity of the subjects under study have been omitted in the text of this article.

### Genomic DNA isolation

DNA was isolated from peripheral blood and tumor samples following standard saline [19] and phenol/chloroform [20] procedures.

### Mutational screening

All patients were previously screened for mutation in *WT1*, *WTX* and exon 3 of *CTNNB1* [21].

### CNV screening by WT-Customized aCGH

We selected 60-mer oligonucleotide features from Agilent’s eArray probe library (Agilent, https://earray.chem.agilent.com/earray) in a customized, high resolution format of 4x180K. This array comprised specific probes covering *WT1*, *WTX*, *CTNNB1*, 16 regions involved in WT and the most relevant microdeletion and duplication syndromes as well as probes for the library backbone. Average probe spacing between regions of interest was 5 kb. Regions reported as non-pathogenic in the Genomic Variant Database (http://projects.tcag.ca/variation/) were excluded from the design to exclude CNV lacking apparent clinical relevance.

Array experiments were performed as recommended by the manufacturer (Agilent Technologies, Santa Clara, CA, USA). Briefly, DNA (500 ng) from the study specimen and a same-sex reference (Yoruba Male NA18507 and Yoruba Female 18517, Coriell Institute, USA) were double-digested with *Rsa*I and *Alu*I for 2h at 37°C. Following heat inactivation of enzymes at 65°C for 20 min, each digested sample was labeled by random priming (Genomic DNA Enzymatic Labeling Kit Agilent) for 2 h with Cy5-dUTP for patient DNA and Cy3-dUTP for reference DNA. Labeled products were purified in columns (Microcon Ym-30 filters, Millipore Corporation). Following probe denaturation and pre-annealing with *Cot*-1 DNA, hybridization was performed at 65°C under rotation for 24 h. After two washing steps, microarrays were scanned with the Agilent Microarray Scanner. Data were extracted with Feature Extraction software (v9.1 Agilent Technologies) and analyzed with Genomic Work Bench 7.0.4.0 and Agilent Cytogenomics 2.7.8.0.

A detailed description of the statistical algorithms is available in the manual provided by Agilent Technologies. The Aberration Detection Method 2 (ADM-2) quality weighted interval score algorithm identified aberrant intervals in samples showing consistently high or low log ratios based on their statistical score. The score represented the deviation of the weighted average of the normalized log ratios from its expected value of zero calculated with Derivative Log2 Ratio Standard Deviation algorithm. A Fuzzy Zero algorithm was applied to incorporate quality information on each probe measurement. Our threshold settings for the CGH analytics software to make a positive call were 6.0 for sensitivity, 0.35 for minimum absolute average log ratio per region, and 5 consecutive probes with the same polarity were required for the minimum number of probes per region.

## Results

In this study, we analyzed CNV with a WT-Customized aCGH platform, using paired, blood/tumor DNA samples from 14 patients with sporadic WT. Our findings, together with clinical and histological data, are shown in Table 1. Raw data were deposited in the GEO repository with the reference GSE69971.

**Fig 1:**
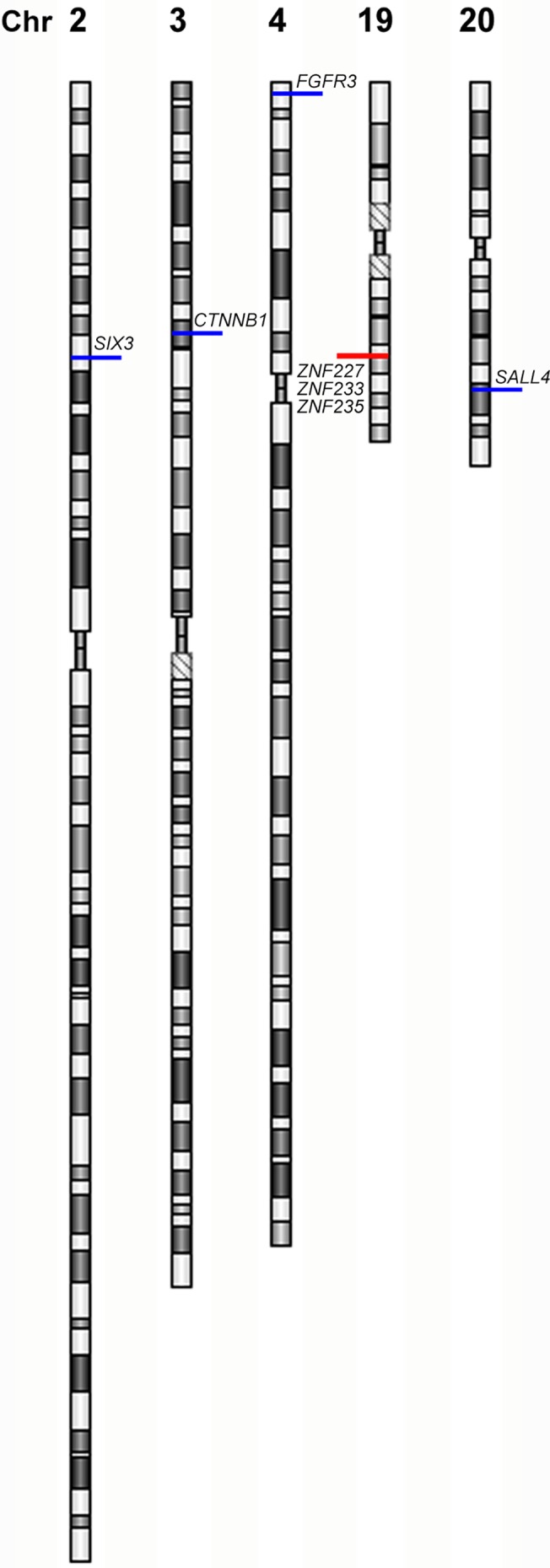
Novel constitutive CNV detected in Wilms Tumor. Schematic representation of CNVs detected in both blood and tissue by aCGH. Duplications (blue bars): 2p21 including *SIX3*, 3p22.1 with *CTNNB1*, 4p16.3 with *FGFR3*, and 20q13.3 with *SALL4*. Deletion (red bar): 19q13.31 including *ZNF227*, *ZNF233* and *ZNF235*. All the alterations were also detected in matched tumor samples.

Gains or losses involving whole chromosomes or chromosome arms were not observed in blood DNA but segmental alterations were observed in 4/14 patients. Gains in 4/14 (28.6%) patients included the following regions within which some genes were identified: 2p21 with *SIX3* (1/14), 3p22.1 with *CTNNB1* (1/14), 4p16.3 with *FGFR3* (1/14), and 20q13.3 with *SALL4* (1/14). A single segmental loss was identified involving 19q13.31 with *ZNF227*, *ZNF233* and *ZNF235* in 1/14 patient. All these alterations were also found in matched tumor samples (Fig 1).

Tumor samples showed whole chromosome gains in 8/14 (57.1%) patients and whole chromosomes losses in 3/14 (21.4%). Arm gains (p or q) were found in 6/14 (42.9%) patients and losses in 4/14 (28.6%). Segmental gains were observed in 9/14 (64.3%) patients and losses in 8/14 (57.1%). Previous data on *WT1*, *WTX* and *CTNNB1* exon 3 mutational screening showed point mutations in four patients (28.6%) [17].

Among the three patients with bilateral WT, a 1q gain in tumor samples was found in Patients 8 and 10. Patients 4 and 8 also presented PLNR, and both of them showed gains of chromosomes 6 and 12. Patient 4 presented several chromosome gains but no losses, while tumor sample from Patient 8 presented one arm loss and fewer gains. Two focal, anaplastic WT (in Patients 4 and 5) showed different extensive gains or losses but both patients showed, in their paired samples, alterations of well-known WT-related genes: *CTNNB1* within a 3p22.1 gain in Patient 4, and a p.R560W hemizygous *WTX* mutation in Patient 5. Chromosome-, arm- or segmental alterations were unrelated to histological profiles and mutations affecting *WT1*, *WTX* and *CTNNB1*.

Novel somatic abnormalities included, among others (Table 1), loss of 2p16.1-p15 including *REL*, *COMMD1*, *EHBP1*, *PAPOLG*, and *PUS10* in Patient 5 with focal anaplasia, loss of 19p13.3 including *GNG7*, *SLC39A3*, *SGTA*, *TLE6*, *TLE2*, *CELF5*, *NFIC*, *FZR1*, *HMG20B*, *GIPC3*, *DAPK3*, *MATK*, *EEF2*, *PIAS4*, *ZBTB7A*, and *MAP2K2* in Patient 1, and gain of the same 19p13.3 region in Patient 12 including different genes (*PALM*, *FSTL3*, *RNF126*, *FGF22*, *POLRMT*, *HCN2*).

## Discussion

In this study, blood and tumor DNA samples from 14 patients with sporadic WT were analyzed for CNV, with the aim of identifying germline and somatic anomalies associated to this tumor. To our knowledge, this is the first aCGH analysis of paired samples of this tumor. CNV were apparently unrelated with the heterogeneous histological profiles of the samples herein studied. Among chromosome abnormalities, 9 of 41 (22%) were novel (Table 1) pointing to a likely role in WT pathogenesis.

All alterations detected in blood were segmental and were also present in their corresponding tumor samples, comprising 4 gains (3p22.1, 4p16.3, 2p21 and 20q13.3) and one loss (19q13.31) in 4/14 (28.6%) patients (Fig 1). Two of these gains, 3p22.1 and 4p16.3, have been previously described as part of larger segmental gains, and only in tumor samples, respectively [6, 22]. Constitutive alterations coexisted with atypical phenotypes unrelated to defined clinical syndromes suggesting their possible association to congenital abnormalities and predisposition to cancer.

Gain of 3p22.1 in Patient 4 included *CTNNB1*, a gene that encodes β-catenin and an essential participant of the Wnt signaling pathway whose deregulation is critical for tumor development. Somatic mutations affecting β-catenin are present in >10% of WT and are frequently associated to the presence of *WT1* mutations [6, 23, 24]. This patient showed dysmorphic features, with triangular face and syndactyly of fingers 2 and 3. Large constitutive gains and losses containing this chromosome region are registered in DECIPHER database v8.6 (Database of Chromosomal Imbalance and Phenotype in Humans using Ensembl Resources) [25] and Database of Genomic Variants (DGV) (http://dgv.tcag.ca/), albeit unrelated to WT. In DECIPHER, complete gains and losses of *CTNNB1* were registered while, in DGV, a duplication including *CTNNB1* was part of a complex rearrangement. These alterations, however, differed from the partial duplication of *CTNNB1* exon 8 and 9 found by us, and thus may have different functional effects. The 3p22.1 gain thus represents a novel alteration associated to WT.

Gain of 4p16.3 in Patient 13 included *FGFR3* exon 11 and part of exon 12. This gene is a member of a receptor tyrosine kinase family regulating cell proliferation, differentiation and migration, and also involved in selective apoptosis during embryogenesis. *FGFR3* mutations had been associated with congenital skeletal disorders and several cancers but not with WT [26]. Large CNVs of this region were registered in ClinVar (http://www.ncbi.nlm.nih.gov/clinvar/)—a database that aggregates information about genomic variation and its relationship to human health—and DECIPHER, which included the complete *FGFR3* gene while, in DGV, deletions accounted only for internal alterations of this gene. The 4p16.3 gain including *FGFR3* exon 11 and part of exon 12 is thus a novel alteration associated to WT.

A constitutive 2p21 gain was also found in Patient 13. Constitutional gains or losses of 2p21 were described in association with a wide range of dysmorphic manifestations, skeletal abnormalities, holoprosencephaly and intellectual disability [27, 28]. One patient, with a larger genomic gain (27.21 Mb) of this region, abnormality of the kidney, intellectual disability and prenatal short stature was reported, albeit without WT [25]. The 2p21 gain reported herein included the terminal region of *SIX3* exon 2. This gene is a regulator of the Wnt signaling pathway [29] whose alterations are associated with holoprosencephaly [30]. *SIX3*, together with *SIX1* and *SIX2*, are members of the transcription factor family *SIX*, with important roles in regulation of cell proliferation, survival, migration and invasion [31]. *SIX1* promotes mesenchymal-to-epithelial transition in different tumor types [32]. On the other hand, embryonic, renal mesenchyme contains pluripotent progenitor cells characterized by expression of *SIX2* that suppresses cellular differentiation [33]. Recently, somatic mutations have been described in a hotspot of the *SIX1* and *SIX2* homeo-domain responsible for DNA binding and protein interaction in WT. Both mutant and wild type *SIX1/2* alleles have been found to be highly expressed, a reason why these mutations were considered to be activating, resulting in failure of mesenchymal-to-epithelial transition and continued proliferation of the metanephric mesenchyme [34, 35]. DECIPHER registered manifold 2p21 CNVs including several genes, among which the complete *SIX3*, unrelated to WT. In ClinVar, two gains of the terminal region of *SIX3* exon 2 were associated to clinically benign conditions. The 2p21 gain reported herein is thus a novel alteration associated to WT.

A 20q13.3 gain, found in a patient with a high number of dysmorphic features (Patient 11), included part of the zinc finger transcription factor *SALL4*, a gene involved in the maintenance and the pluripotency of embryonic stem cells. This gene has been found to be expressed in WT and developing fetal kidney [36] while *SALL4* deletions are a common cause of Okihiro, acro-renal-ocular, and *SALL4*-related Holt-Oram syndromes [37], all of which associated with renal morphological malformations rather than WT. In DECIPHER and ClinVar, 20q13.3 CNVs were not associated to WT but rather to developmental delay and cardiac abnormalities while, in DGV, a complete *SALL4* loss has been registered. The 20q13.3 gain herein reported is thus a novel anomaly associated to WT, suggesting that *SALL4* might be relevant for the genesis of this tumor.

Loss of 19q13.31 in Patient 6 included three zinc finger protein genes (*ZNF227*, *ZNF233* and *ZNF235*), which could not be associated with clinical manifestations. Although this loss has not been previously associated with WT, functional studies of these zinc finger genes in WT might provide future insights on their role in tumor development. In Clinvar, one 19q13.31 duplication associated to cardiac alterations has been reported while, in DECIPHER, deletions of this region have not been registered. In DGV, CNVs did not include the three genes involved in the 19q13.31 loss reported herein. This makes this loss a novel alteration associated to WT.

Several previous studies in sporadic WT reported whole gains of chromosomes 12, 8, and 6, gains of 1q, losses of 16q, and segmental rearrangements involving chromosomes 1, 7, and 17 in tumor tissue [13, 38–42]. In this study, several CNV were frequently found in tumors samples, like gain of 1q and 16p as well as loss of 7p and 16q. Novel segmental somatic alterations comprised gain of 19p13.3 and 20p12.3 and loss of 2p16.1-p15, 4q32.5-q35.1, 4q35.2-q28.1 and 19p13.3.

A strong association has been described between gain of 1q and loss of 1p (P<0.001), and between gain of 1q and loss of 16q (P<0.001), this latter reflecting the occurrence of a typical t(1;16) [14]. Somatic gain of 1q21-q25 was associated with poor prognosis in WT patients with favorable histology [17]. In this study, all segmental gains including 1q21-q25 occurred in tumor samples. Loss of 1p, one of the most frequent chromosome alterations in WT samples and crucial for tumor development [13, 40–42], was not found in our patients. However, a small segmental loss (1p22.2-p36.3) was observed in one case of stage V, bilateral, triphasic WT, suggesting that this loss might be associated with tumor progression rather than carcinogenesis.

The two deleted regions including 4q (4q32.5-q35.1 in tumor sample from Patient 14 and 4q35.2-q28.1 in tumor sample from Patient 8) contained several genes, including *NEK1*. This gene encodes a protein involved in cilium control and is essential for response to DNA damage, cell cycle progression, and correct transmission of chromosomes to daughter cells. *NEK1* is developmentally regulated and strongly expressed in kidney, mainly in podocytes and tubular epithelial cells. *NEK1* deficiency causes early abnormalities in kidney development, including excessive apoptosis and diminished proliferation, and has been associated with polycystic kidney disease [43]. On the other hand, increased *NEK1* expression is associated to a decreased sensitivity to treatment of damaged DNA in renal cell carcinoma [44]. Patient 8 also carried a somatic *CTNNB1* mutation, while Patient 14 showed a somatic duplication of chromosome 3 where this gene is located. Patient 4 carried a constitutive and a somatic 3p22.1 duplication, in agreement with previous studies showing gain-of-function, *CTNNB1* mutations in WT [45]. *NEK1* and *CTNNB1*, together with *AXIN1*, *GSK3B* and *TSC2*, participate in the Wnt signaling pathway in urological cancer cells and polycystic kidney disease (PKD) [46, 47]. Whether loss of *NEK1* might coexist with β-catenin stabilization, similarly to *WT1* alterations, for the survival and proliferation of tumor cells [45] is an open question. This might be clarified by analyzing the expression profiles of *CTNNB1*-mutants versus *CTNNB1* wild-type in WT with *NEK1* alterations.

Chromosome region 11p13 was the first locus implicated in the development of WT whose alterations have been detected both by karyotyping and DNA arrays [48]. This region contains *WT1*, the first reported gene associated to the etiology of WT [49, 50], which is affected by deletions and point mutations in 10–20% of WT [51, 52]. *WT1* encodes a transcription factor that is essential for survival and differentiation of renal cells and subsequent kidney development. Additionally, it has both tumor suppressor and oncogenic activities [53] while its epigenetic silencing can also contribute to sporadic WT [54]. In our study, one 11p13 gain and one loss were found in tumor samples from Patients 8 and 1, respectively.

Rearrangements encompassing 17p may result in loss of *TP53* (in 17p13.1), resulting in alterations of cell cycle checkpoint controls, apoptosis, cell migration and invasion. Secondary to *TP53* loss, structural rearrangements in 17q are strongly associated with an unfavorable anaplastic histology [39, 40, 42]. In our series, however, a WT with a favorable histology (in Patient 3) showed a novel segmental loss in 17p13.2-p11.2. Also, a novel structural somatic gain of 17q21.31-q25.3 was observed in Patient 8 presenting bilateral WT, PLNR, and histology with epithelium predominance. This histological profile was coincident with the previous finding that tumors originated from PLNR usually present a predominance of epithelium or blastemal components [55, 56].

Several genes associated with carcinogenesis, but not with WT, are present in 2p16.1-p15 and 19p13.3. Patient 1 showed a somatic 19p13.3 segmental loss including *PIAS4*, a regulator of cellular senescence and apoptosis through activation of p53-mediated cell death, a pathway also positively regulated by WTX [57]. Patients 4 and 5 showed a 20p12.3 somatic duplication. This region contains *BMP2*, a gene that belongs to the transforming growth factor-beta (TGFB) superfamily. It is involved in mesenchymal progenitor cell chemotaxis through PI3K signaling induction [58] and has been identified as an epigenetically silenced growth inhibitor in rhabdomyosarcoma [59]. Furthermore, it also inhibits hepatocellular carcinoma growth and migration through down-regulation of the PI3K/AKT pathway [60]. As the RING domain of *PIAS4* is also involved in the suppression of the BMP-signaling pathway [61], the *BMP2* duplication carried out by Patients 4 and 5 might produce similar effects to the loss of *PIAS4* in Patient 1.

To discard the new changes described in this study are small, previously undocumented CNVs that may also be present in the constitutional DNA of control individuals, in addition to public databases we have compared them with 3500 pseudo-controls studied in our Centre with a related customized aCGH [62] covering the regions included in 5 of the 9 new described CNVs. In none of the cases these alterations were detected. These suggests that these CNVs are associated with WT in our sample, and further studies should clarify whether they are recurrent and directly associated with carcinogenesis or if instead they are passenger alterations neutral to tumor cell selection.

To our knowledge, this is the first study using aCGH for detecting chromosomal alterations in paired blood and WT tumor samples. Although several of these alterations may not be recurrent but rather passenger alterations restricted to our sample, the novel chromosome abnormalities might be contributing to carcinogenesis, pointing to several candidate genes that might be constitutively (i.e. *SIX3*, *SALL4*) or somatically involved (i.e. *NEK1*, *PIAS4*, *BMP2*) in WT development and progression.
